# Cancer obscures extrapulmonary tuberculosis (EPTB) at a tertiary hospital in Northern Malawi

**DOI:** 10.1017/S095026882000254X

**Published:** 2020-10-20

**Authors:** M. R. O. Chisale, F. Sinyiza, P. Kaseka, J. S. T. Wu, C. Chimbatata, B. C. Mbakaya, P. S. Kamudumuli, A. B. Kayira

**Affiliations:** 1 Mzuzu University, Faculty of Sciences, Technology and Innovations, Biological Sciences, P/Bag 201 Luwinga, Mzuzu, Malawi; 2 Ministry of Health, Mzuzu Central Hospital, P/Bag 209, Luwinga, Mzuzu, Malawi; 3 Luke International, Mzuzu, Malawi; 4 Pingtung Christian Hospital, Overseas Services, Taipei, Taiwan; 5 St Johns Institute for Health, Mzuzu, Malawi; 6 URC Malawi Laboratory Project, Mzuzu, Malawi

**Keywords:** Extra-pulmonary tuberculosis (EPTB), tuberculosis (TB), Malawi, cancer, obscures

## Abstract

Data on the prevalence of extrapulmonary tuberculosis (EPTB) patients are limited in many African countries including Malawi. We conducted a retrospective review of all histology reports for cancer suspected patients at Mzuzu Central Hospital (MZCH) between 2013 and 2018 to determine the proportion of EPTB cases among cancer suspected patients and characterised them epidemiologically. All reports with inconclusive findings were excluded. In total, 2214 reports were included in the review, 47 of which reported EPTB, representing 2.1% (95% CI 1.6−2.8). The incidence of EPTB was significantly associated with sex, age and HIV status. Men were more than twice (OR 2.1; 95% CI 1.2–3.9) as likely to have EPTB as women while those with HIV were more than six times (OR 6.4; 95% CI 1.7–24.8) as likely to have EPTB compared to those who were HIV-negative. EPTB demonstrated an inverse relationship with age. The highest proportion of EPTB was found from neck lymph nodes (10.3% (5.4–17.2)). A reasonable number of EPTB cases are diagnosed late or missed in Malawi's hospitals. There is a need for concerted efforts to increase EPTB awareness and likely come up with a policy to consider EPTB as a differential diagnosis in cancer suspected patients.

## Introduction

Tuberculosis (TB) is a disease which remains a big challenge to be conquered in countries with a high burden of TB. Although the literature shows that pulmonary TB is the most common presentation of TB disease globally, it can also involve any organ in the body [1]. Extrapulmonary tuberculosis (EPTB) is defined as the occurrence of TB in any part of the body other than the lungs [1, 2]. Manifestations may relate to the system involved, or simply as prolonged fever and non-specific systemic symptoms, hence diagnosis may be elusive and is usually delayed [3].

The proportion of EPTB among TB cases varies from country to country. In Malawi, WHO estimates indicate that the general TB incidence has been significantly reducing from 50 000 cases per year (332 cases per 100 000 persons) in 2010 to 29 000 cases per year (159 cases per 100 000 persons) in 2016 [4, 5]. However, the incidence in EPTB has not been comparably reduced as there have been fluctuations in the incidence rates. For instance, in 2010, WHO estimates show that Malawi recorded 4857 EPTB cases, while in 2011, there were 5076 cases. Since then, there has been a slight decrease to 4093 in 2016 [4, 5]. This shows that while there might be a huge decrease in PTB, the same may not be true for EPTB. Some studies have cited misdiagnosis challenge as one of the key issues which has hampered the success in the fight against EPTB due to its non-specific signs and symptoms unlike in PTB [2, 6, 7].

Diagnostic challenges and the burden of HIV are the major contributing factors for between-country and in-country variation of EPTB. However, when clinically suspected, EPTB can easily and rapidly be diagnosed using several laboratory methods including histological, lateral flow assay, molecular methods (GeneXpert), culture and microscopy [8, 9]. Malawi, and Mzuzu Central Hospital (MZCH) in particular, has all of these tools at its disposal except for histology which it procures from a private laboratory for cancer suspected patients, the results of which take at least a month to arrive. Studies conducted by the WHO and others have indicated that TB culture is as good as histology for diagnosing EPTB [3, 8–11]. MZCH laboratory is therefore fully able and capable of providing a rapid diagnosis of EPTB whenever it is suspected.

However, while reviewing 5 years data (2013–2018) of biopsies sent for histological analysis to confirm or exclude cancer at MZCH, we discovered that a good number of the cases turned out to be EPTB. It was from this background that we got curious and sought to comprehensively review all histological results (2013–2018) of cancer suspected patients at MZCH in order to determine what proportion of these yielded EPTB and further characterise (based on epidemiological factors such as gender, age, HIV status and specimen sites) the EPTB cases identified.

## Methodology

### Study design

This was a record-based retrospective study involving a cohort of histopathology reports of cancer suspected patients from 2013 to 2018.

### Setting

The study was conducted at MZCH which is situated in the Northern Region of Malawi.

Malawi is a landlocked country and has a population of around 17 506 022, with 13% of the population living in the northern region [12]. The northern region has a population density of 18.4/km^2^. The Malawi Government provides free health care services to all its citizens with a minimal private sector and mission hospitals involved in the provision of health care service, including TB services. The Malawi Government declared TB as an emergency in 2007 [13]. All programmes which involve TB care services in Malawi are operated by the National TB Control Program (NTP) which has adopted and implemented the WHO Stop TB strategy and has incorporated it in its development strategy [13, 14]. The following are the pillars of NTP's activities which are in line with the WHO STOP TB Strategy; pursuing high-quality DOTS expansion and enhancement, addressing TB/HIV, MDR-TB and other challenges and engaging all care providers.

MZCH is the only referral facility in the northern region. Although MZCH is the only facility that provides tertiary care in the region, the hospital has no pathology laboratory. As such, all histology samples are sent to a private pathology laboratory situated in the central region of Malawi which is about 350 km from MZCH.

### Study participants

This study reviewed cancer suspected patient histology reports biopsy specimens which were sent and analysed at pathology laboratory in Lilongwe which serves as the MZCH pathology referral laboratory. The reports reviewed were from 2013 to 2018. Trained data collectors retrieved histopathology reports from MZCH laboratory archives. This study used census approach where all the available histopathological records were included excluding the records with inconclusive results.

### Sample analysis flow

**Fig. 1.**
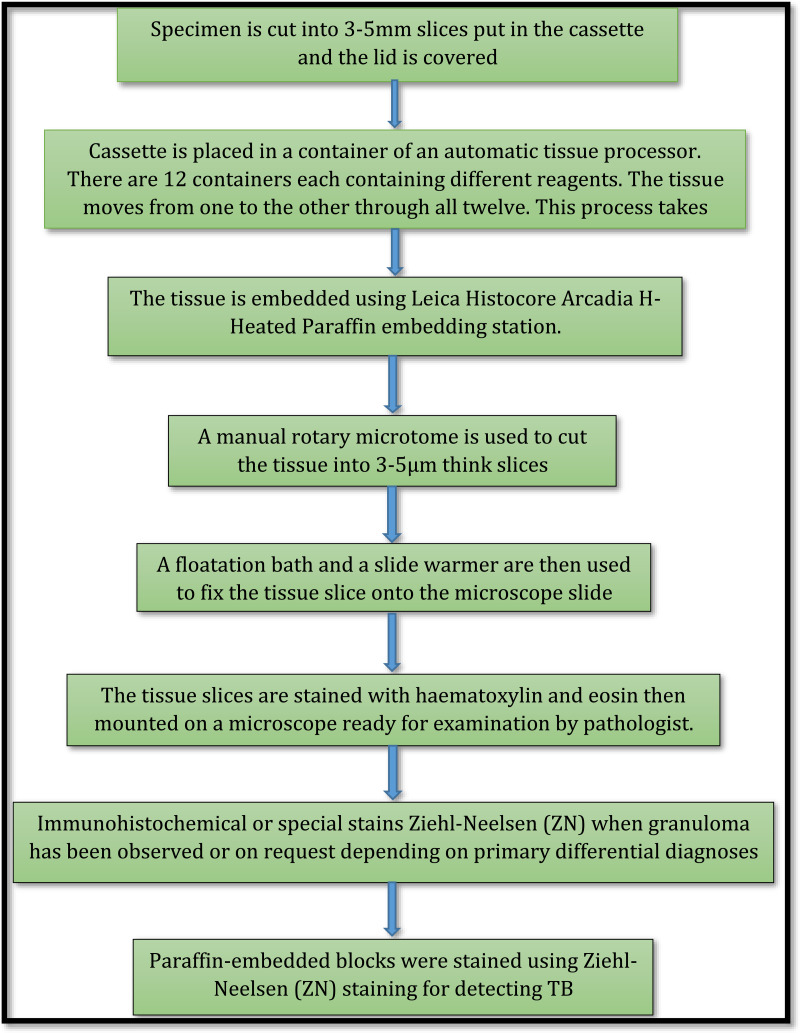
Processes involved in specimen processing at histology laboratory.

Biopsy samples collected from various departments at MZCH are received at the MZCH laboratory reception. Then they undergo a pre-analytical process which includes checking the sample quantity, labelling and ensuring that all necessary documentation has been completed. Samples are then shipped to the referral laboratory using a courier service. At the pathology laboratory, samples are processed and analysed in a stepwise manner as shown in Figure 1. As indicated in Figure 1, the decision to consider testing for TB arrives when the pathologist has observed a granuloma (a distinct granulation tissue is produced in response to an infection/inflammation; it is different from that of surrounding tissues and it is multinucleated). However, the granulomas are also seen in other conditions, hence there is a need for special stain called Ziehl–Neelsen (ZN) test to rule out TB. After the stain, the AFBs were seen clearly from the ZN slides.

### Data entry, analysis and statistics

Data were entered into Microsoft excel 2016, validated and cleaned before importing into Stata, version 13.0 (Stata Corp. LP, College Station, TX, USA) for analysis. Descriptive analyses were performed to summarise patients’ demographic and clinical characteristics, and histopathology results. A multivariate logistic regression was used to assess the risk factors for EPTB.

### Ethics

Ethical approval for the study was obtained from the National Health Science Research Committee (NHSRC) with approval number 19/05/2316. Permission to conduct the study was obtained from MZCH authority.

## Results

In total, 2214 histology reports were reviewed. Forty-seven (47) of these yielded EPTB, representing 2.1% (95% CI 1.6–2.8%). As shown in Table 1, the proportion of EPTB was significantly higher among men than women (OR 2.1; 95% CI 1.2–3.9). EPTB showed an inverse relationship with age, decreasing substantially with increasing age. Patients aged between 41 and 60 years and those over 60 years of age were 70% (OR 0.3; 95% CI 0.1–0.8) and 90% (OR 0.1; 95% CI 0.0–0.5), respectively, less likely to have EPTB compared to those aged 20 years and below. When it comes to HIV status, the proportion of EPTB was more than six times higher (OR 6.4; 95% CI 1.7–24.8) among patients who were HIV-positive compared to those who were HIV-negative.

**Table 1.** Association between demographic characteristics of patients and incidence of EPTB

Variable	Number of biopsies	Frequency of EPTB	Percentage (95% CI)	Unadjusted odds ratio (95% CI)	*P*-value	Adjusted odds ratio (95% CI)	*P*-value
Sex							
Female (Ref.)	1423	25	1.8 (1.1–2.6)				
Male	737	22	3.0 (1.9–4.5)	1.8 (1.0–3.2)	0.05	2.1 (1.2–3.9)	0.014
Undocumented	53	0	0 (0.0–6.7)				
Age (years)							
1–20 years (Ref.)	317	12	3.8 (2.0–6.5)				
21–40 years	800	24	3.0 (1.9–4.4)	0.8 (0.4–1.6)	0.504	0.8 (0.4–1.7)	0.609
41–60 years	671	9	1.3 (0.6–2.5)	0.3 (0.1–0.8)	0.017	0.3 (0.1–0.8)	0.018
>60 years	379	2	0.5 (0.1–1.9)	0.1 (0.0–0.6)	0.009	0.1 (0.0–0.5)	0.006
Undocumented	46	0	0.0 (0.0–7.7)				
HIV status							
Negative (Ref.)	497	3	0.6 (0.1–1.8)				
Positive	197	8	4.1 (1.8–7.8)	7.0 (1.8–26.6)	0.004	6.4 (1.7–24.8)	0.007
Unknown	1520	36	2.4 (1.7–3.3)	4.0 (1.2–13.0)	0.022	3.3 (1.0–10.8)	0.05

Univariate and multivariate logistic regressions were used to calculate the unadjusted and adjusted odds ratios, respectively.

Table 2 shows that the proportion of EPTB has been fluctuating, first increasing from 3% in 2013 to 3.6% in 2014 before slumping back to 2.4% in 2015, and then continued in that downward trend to 0.9% in 2018.

**Table 2.** The trend in numbers and proportions of EPTB over the studied period

Year	Number of biopsies	Frequency of EPTB	Percentage (95% CI)
2013	406	12	3.0 (1.5–5.1)
2014	84	3	3.6 (0.7–10.1)
2015	370	9	2.4 (1.1–4.6)
2016	591	13	2.2 (1.2–3.7)
2017	419	7	1.7 (0.7–3.4)
2018	344	3	0.9 (0.2–2.5)

When it comes to the distribution of EPTB by site, the highest proportion (10%; 95% CI 5.3–17.2) of cases were diagnosed from neck lymph nodes samples (Table 3).

**Table 3.** Distribution of EPTB cases by sites

Specimen site	Number of biopsies	Frequency of EPTB	Percentage (95% CI)
Breast	202	1	0.5 (0.01–2.7)
Cervix	500	4	0.8 (0.2–2.0)
Neck lymph-nodes	117	12	10.3 (5.4–17.2)
GIT	165	1	0.6 (0.02–3.3)
Oral	52	3	5.7 (1.2–15.9)
Penis	31	1	3.2 (0.1–16.7)
Bone	25	4	16.0 (4.5–36.1)
Mandible	77	2	2.6 (0.3–9.1)
Omentum	9	1	11.1 (0.3–48.2)
Scrotum-testis	17	3	17.6 (3.8–43.4)
Pancreas	1	1	100.0 (2.5–100.0)
Groin	4	1	25.0 (0.6–80.6)
Caecum	4	1	25.0 (0.6–80.6)
Parathoracic vertebra	1	1	100.0 (2.5–100.0)
Other lymph nodes	62	7	11.3 (4.7–21.9)
Others (unnamed area/unspecified site)	316	4	1.3 (0.3–3.2)

## Discussion

We sought to determine the proportion of EPTB among cancer suspected patients at a tertiary hospital in Malawi. We found that 2.1% of cases which clinicians thought it was cancer were actually EPTB. This means that 2.1% of EPTB cases are either being missed or diagnosed late in Malawi's current health system. This is a large number considering that the current national TB pick up rate (pulmonary and extrapulmonary combined) is between 8% and 10%. Furthermore, the actual EPTB rates and case fatality are not clearly specified and emphasised in most cases [15]. It is likely that these cases are eclipsed by other conditions – cancer being the more likely culprit. The lack of specific signs and manifestations which may give a clue to both patients and doctors to suspect EPTB is partly to blame [2, 7, 16–19]. But more so is the increased cancer awareness in Malawi [20–23] which has skewed the clinical judgement of medical practitioners towards cancer.

This study has established that EPTB is more common in males (3.0%) than females (1.8%). This is similar to studies conducted in African countries and elsewhere which found that men are more affected than women [7–9, 15, 24, 25]. The possible explanation to this similarity finding could be due to resemblance in health-seeking behaviour in our set up whereby females seek medical care more frequently and earlier than males, hence posing more chance for male to be a highly affected group of people [26, 27]. The age group 1–20 years was the most affected, constituting a proportion of 3.8% of EPTB cases. This finding is different from other studies conducted in Malawi and elsewhere [7, 9, 15, 24], as many studies show that majority of EPTB patients are within the age range of 20–50 years. This is so because EPTB distribution commonly follows the same pattern as the HIV seroprevalence among the population [15, 28]. However, our findings on age could be due to the fact that cancer is not common among such a population. This could likely tip the physician to consider EPTB first when such populations have a tumour or anything worthy to suspect cancer.

In this study, there was an observation of inadequate data documentation, especially on HIV status such that majority, 76.6% had their HIV status unknown. EPTB was far more common among HIV-positive patients (4.1%) than among HIV-negative patients (0.6%). It is more likely that among the HIV unknown EPTB cases, the majority were HIV-positive [9].

Just like many other studies, the highest number of EPTB cases (40%) were diagnosed from lymph biopsies [1, 2, 7, 16, 18, 24, 29]. It is also worth noting that this study has highlighted other sites (cervix, penis and testis) which are less atypical in the sense that, to the best of our knowledge, no study reported these sites with regard to EPTB. Being such rare sites for EPTB, it is very likely that clinicians would almost always think of cancer whenever confronted with a lesion from these sites.

Besides the sites reported in this study, other research studies have revealed many more sites, of which some are very uncommon such as spine, CNS, skin, pericardium, etc. [1, 2, 9, 15, 16, 18, 29, 30]. This simply confirms that EPTB is indeed a multi-organ disease. There is a need to have policies and concerted efforts aimed at improving the EPTB diagnosis considering that it mimics cancer in most settings due to its non-specific systemic symptoms [20–22], and make it difficult to differentiate these two conditions. Otherwise, EPTB will remain elusive and diagnosis will often be delayed [6]. This is supported by several studies [2, 6, 7] which have cited misdiagnosis challenge as one of the key issues which have derailed efforts in the fight against EPTB.

## Limitations

The following limitations were identified in this study: first, the study was conducted at one tertiary hospital in northern Malawi. As such, the findings cannot be generalised. Second, this study was a record-based retrospective cohort study. Therefore, it was impossible to probe and identify some of the missing variables that would have been significant to this study but were missing on the patients’ files such as HIV status. Nevertheless, this study has provided awareness on how cancer obscures EPTB in Malawi, which could be used to inform practitioners and policy makers.

## Conclusions

It is clear that a substantial number of EPTB cases are either missed or diagnosed late in Malawi's hospitals, perhaps because clinicians tend to suspect cancer more on every tumour/lesion in their clinical practice. This calls for urgent improvements in the diagnosis of EPTB from both the clinical and laboratory fronts. We believe this can be achieved through increasing awareness of EPTB and its clinical presentation/manifestation among physicians. Where possible, it is also important to consider employing TB screening tests to all those with undefined lesions and being suspected of cancer. There is also a need for concerted efforts to increase EPTB awareness and come up with a policy to consider EPTB as a differential diagnosis in cancer suspected patients.

## Availability of data and materials

All data related to this study can be accessed at Mzuzu Central Hospital through the hospital authorities and the corresponding author.
